# Evaluation of the SF-3000 haematology analyser

**DOI:** 10.1155/S146392469600017X

**Published:** 1996

**Authors:** Izumi Tsuda, Hitoshi Sagawa, Takayuki Takubo, Seiki Kawai, Noriyuki Tatsumi

**Affiliations:** Department of Clinical and Laboratory Medicine Osaka City University Medical School 1-5-7 Asahimachi Abeno Osaka 545 Japan


Journal of Automatic Chemistry, Vol. 18, No. 5 (September-October 1996), pp. 163-167

### Evaluation of the SF-3000 haematology analyser

**Izumi Tsuda, Hitoshi Sagawa, Takayuki Takubo, Seiki Kawai and Noriyuki Tatsumi**

*Department of Clinical and Laboratory Medicine, Osaka City University Medical School, 1-5-7 Asahimachi, Abeno, Osaka 545, Japan*

*This paper describes an evaluation of newly developed SF-3000 haematology analyser. This instrument is smaller than comparable instruments, and uses a semiconductor laser. Its precision and reproducibility were found to be good. Accurate measurements of the leukocyte differential were obtained from stored blood (kept at room temperature and 4°C) for up to 24 hours after collection. A good correlation was found between the SF-3000 and SE-9000 haematology analyser for the red cell and platelet parameters on the 246 samples tested. The leukocyte differential produced by the SF-3000 correlated well with the authors' routine method, with $r^2$ values over 0·9 for the percentages of neutrophils, lymphocytes and eosinophils .*

### Introduction

Until recently, automatic haematology analysers with a white cell differential function were used only in relatively large laboratories. This is because they were expensive and were designed to process a large number of samples. The reliability and usefulness of these instruments have been well recognized among users.

A new haematology analyser, the SF-3000 (from Sysmex), which is capable of five-part differentials, was designed for use in smaller laboratories. The SF-3000's differential counting technology is unique because it uses a semi-conductor laser. Because the semiconductor laser is compact, it is smaller than other instruments with similar functions. This paper reports on the performance of the SF-3000 analyser compared with the author's routine method.

### Materials and methods

Venous blood treated with K2-EDTA was processed within 6 h (except for the stability tests). The samples were selected from the routine workload, except where stated. The samples were processed on the laboratory's routine haematology analyser (SE-9000 [1], Sysmex-Toa Medical Electronics, Kobe, Japan). The standard protocol for the differential counts in the authors' laboratory was to first use the SE-9000 to screen for abnormal samples, and then to perform a manual differential on these flagged abnormal samples. The samples obtained from patients with a known haematological disease were sent for a manual differential, regardless of the results obtained using the SE-9000. Manual differential counts were performed on May Grünwald-Giemsa stained peripheral blood films prepared by the spinner method. Medical technologists with more than 10 years of experience performed 100 cell differential counts. For the comparison of the differential counts, samples were selected to include a similar percentage of morphologically normal and abnormal samples. All abnormalities were confirmed by the manual method.

### Determination principle

A flow chart of the measurements is shown in figure 1. The sizing and counting of the red blood cells and platelets in the SF-3000 are based on the impedance principle, while haemoglobinometry is based on the sodium lauryl sulphate method [2] in order to eliminate hazardous cyanide waste. These determination methods are the ones used for the Sysmex instruments, including the SE-9000.

Two channels, a DIFF channel and a WBC/BASO channel, are used for the differential counts of white blood cells, and both channels use a semiconductor laser. In each channel, 33 µl of whole blood are mixed with specific reagents (or a single reagent) at 35°C, and then sent to a flow cell. The red blood cells are rendered invisible to the laser. A sheath stream flow-cell focuses the samples. As the white blood cells enter the laser beam, their light scatter is measured at low angles (1–6 degrees) and high angles (8–20 degrees). At the low angle scatter, information on the cell volume is obtained, and at the high angle scatter, intracellular information is obtained.

In the DIFF channel, blood is mixed with two reagents to stabilize the morphology of the white blood cells and haemolyse the red blood cells. White blood cells retain their size in these reagents, except for the monocytes which shrink a little. In this channel, the white blood cells are differentiated into four clusters: lymphocytes; monocytes; neutrophils and basophils; and eosinophils (see figure 2). In the WBC/BASO channel, all cells except for the basophils shrink due to the acid reagent. The basophils are located on the upper side of the scattergram. The actual scattergram displays each white cell population in a distinct colour.

### Results

### *Stability of the differential of white blood cells*

Five samples from normal donors were stored at room temperature (20°C) or at 4°C, and measured 1, 4, 8, 12, and 24 h after blood collection. The samples stored at 4°C were left at room temperature for 10 min before measurement. Three measurements were performed on each sample, and the mean value was used for the analysis.

0142-0453/96 $12.00 © 1996 Taylor & Francis Ltd.

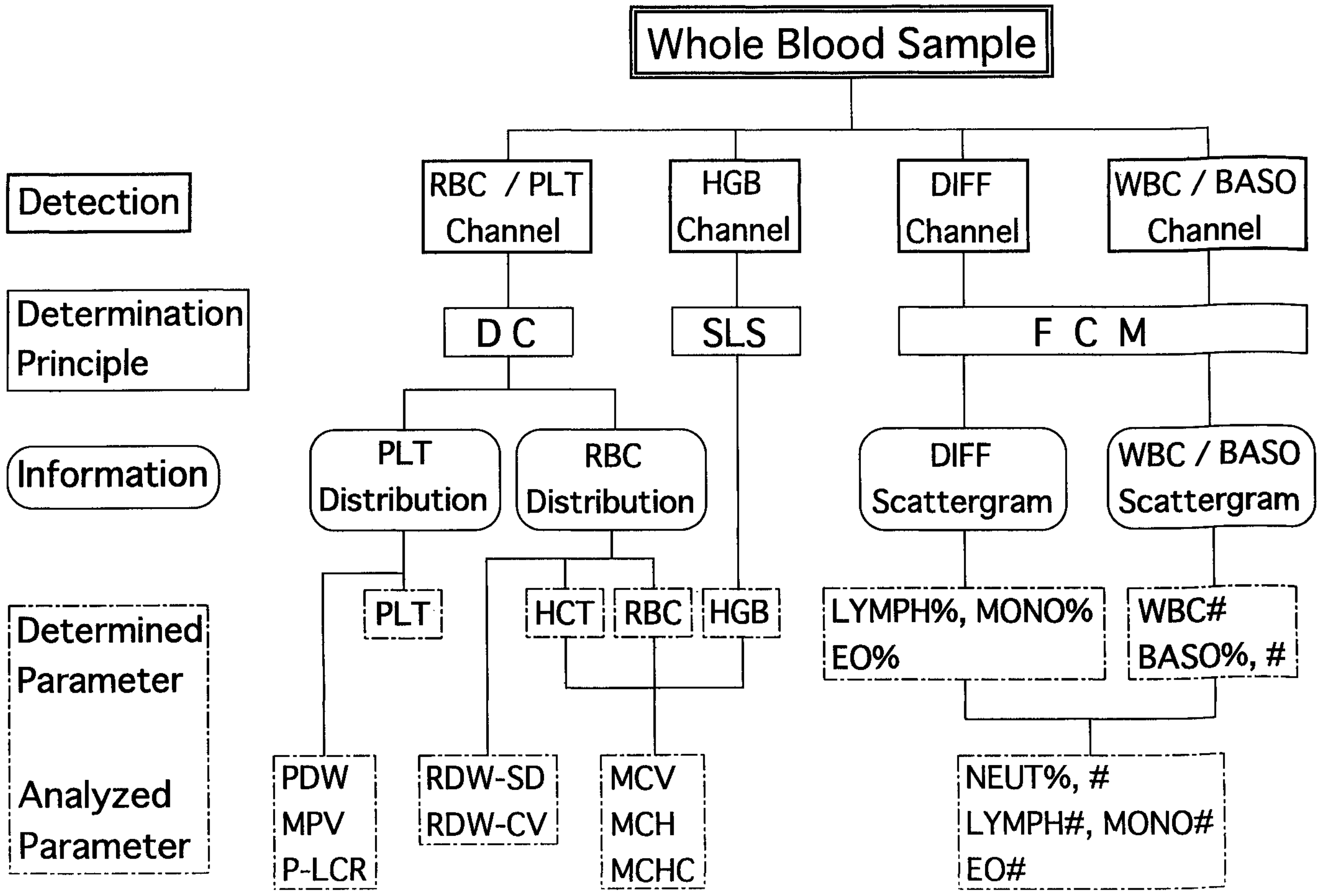

*Figure 1. Flow chart of the analysis performed by the SF-3000. Where DC = direct currency; SLS = sodium lauryl sulphate method; and FCM = flow cytometry.*

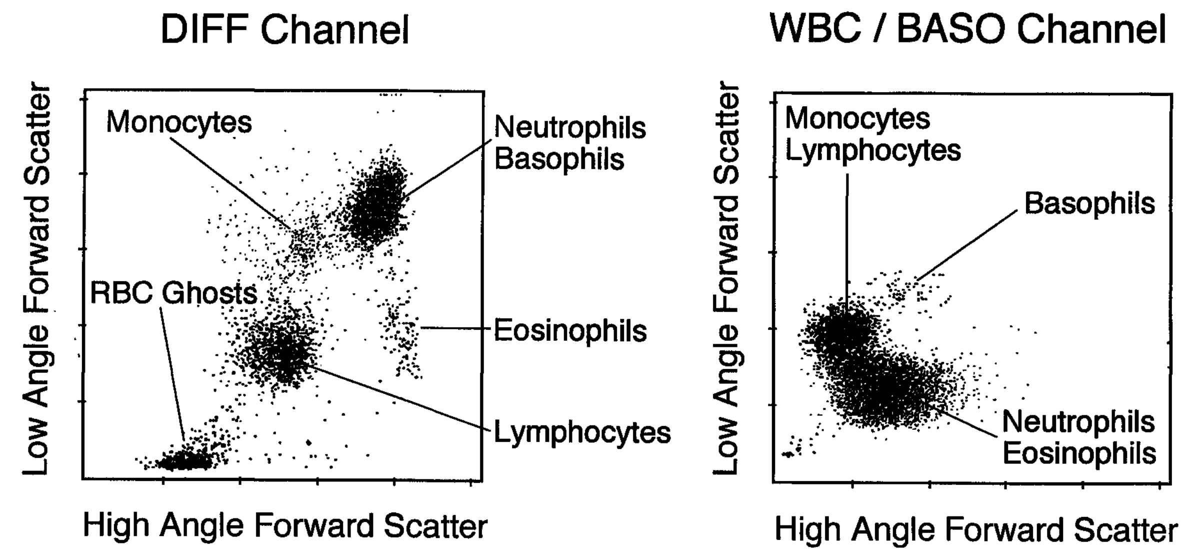

*Figure 2. Scattergrams of DIFF channel (left) and WBC/BASO channel.*

The percentage changes in the differential percentages of the white blood cells were calculated for each sample; the value at 1 h was set at 100%. The changes up to 12 h were slight, and the changes in percentage monocytes and percentage basophils increased after 24 h of storage (figure 3). However, the actual changes in the percentages of the differentials of such cells were less than 2% even at 24 h. No effect of temperature on the stability was observed.

*Reproducibility*

Samples from five normal donors were each analysed 10 times. The measurements were performed within 1 h and

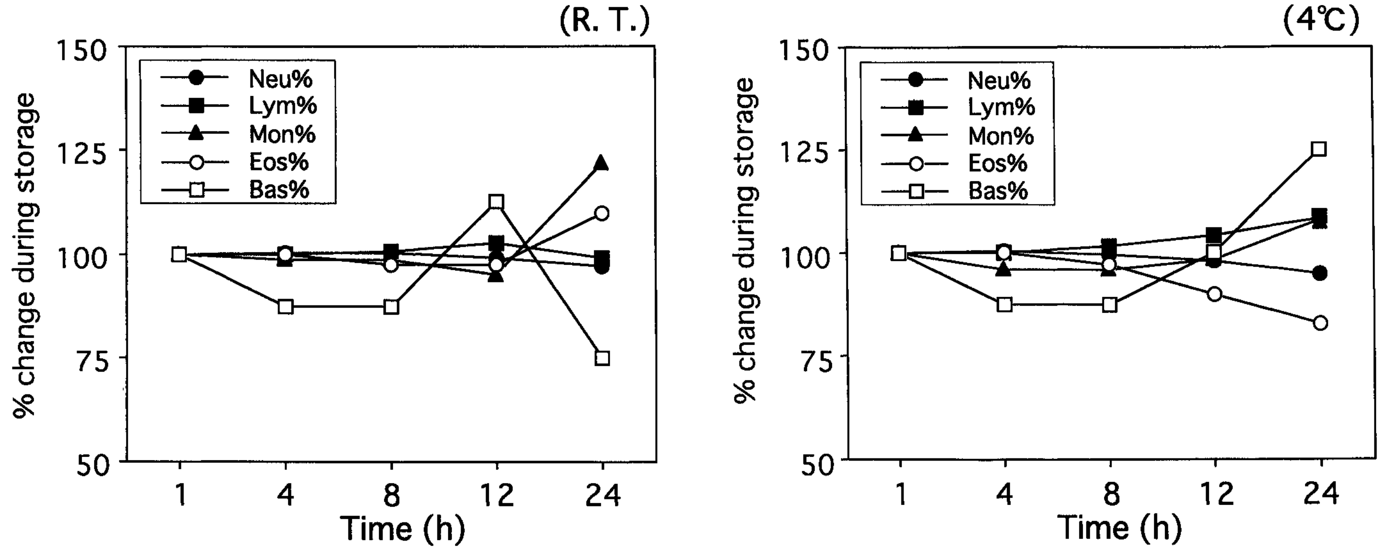

*Figure 3. Percentage changes in white cell differential during storage at room temperature (left part) and 4°C (right part). Each point represents the mean of 10 samples.*

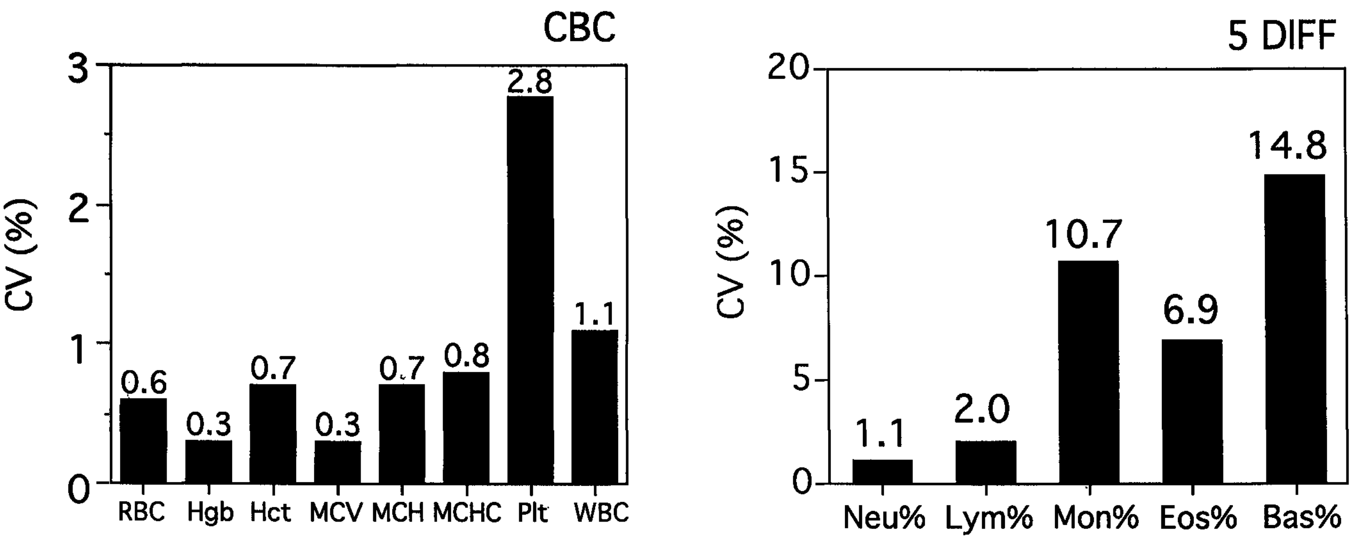

*Figure 4. Reproducibility of complete blood count and the white cell differential. Each bar shows the mean of five samples.*

24 h after the collection of the blood. The mean CV for each parameter is shown in figure 4. The CVs for the complete blood counts varied between 0·3 and 2·8%. For the differential percentages, the CVs ranged between 1·1% for neutrophils and 14·8% for basophils. The results for samples stored for 24 h showed similar results.

*Linearity*

Seven dilutions were prepared from normal donor blood by mixing with the diluent used for SF-3000. The white blood cell count, red blood cell count, and platelet count decreased with increasing dilution (see figure 5). Linearity was observed in the ranges of 9·9–844·4 ( $\times 10^2/\mu l$) for the white blood cell count, 12–744 ( $\times 10^4/\mu l$) for the red blood cell count, and 1·7–67·6 ( $\times 10^4/\mu l$) for the platelet count.

*Comparison of the SF-3000 and SE-9000 blood counts*

The correlations for the complete blood counts between the SF-3000 and SE-9000 analysers are shown in table 1. They ranged from 0·780 to 0·989.

*Comparison of SF-3000 and the routine differential counts*

Data from the SF-3000 and by the routine method were compared on 118 samples. The correlations between the two methods were good (figure 6).

### Discussion

The most novel feature of SF-3000 haematology analyser is that it uses a semiconductor laser as its light source.

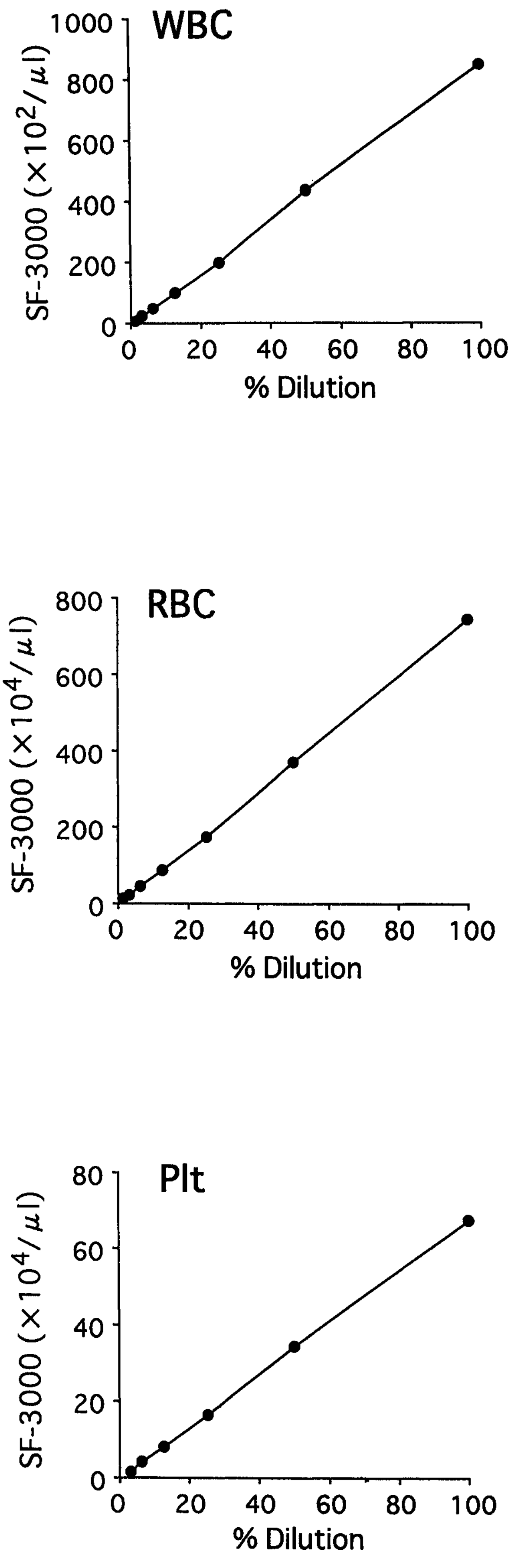

*Figure 5. Linearity for white blood cell count (WBC), red blood cell count (RBC), and platelet count (Plt).*

*Table 1. Correlation of the complete blood count between the SF-3000 and SE-9000 ($N = 246$).*

| Parameter | Correlation | Regression line |
|---|---|---|
| WBC | 0·989 | $y = 0{\cdot}974x + 0{\cdot}878$ |
| RBC | 0·894 | $y = 1{\cdot}006x - 8{\cdot}002$ |
| HGB | 0·905 | $y = 1{\cdot}022x - 0{\cdot}237$ |
| HCT | 0·892 | $y = 1{\cdot}036x - 1{\cdot}058$ |
| MCV | 0·980 | $y = 0{\cdot}993x + 2{\cdot}407$ |
| MCH | 0·976 | $y = 1{\cdot}008x + 0{\cdot}253$ |
| MCHC | 0·780 | $y = 0{\cdot}939x + 1{\cdot}946$ |
| PLT | 0·966 | $y = 1{\cdot}022x + 0{\cdot}283$ |

There are two principles for the enumeration of blood cells and the classification of white cells: the impedance method and the optic method. A helium–neon laser is used as the light source for most optic methods [3, 4] because it can provide strong light even in the very thin light bundle focused to the size of the blood cells. However, these lasers have a relatively short life and require high power. In comparison, the semiconductor laser has a longer life and requires a voltage of only 1–2 V, so its efficiency is good. Furthermore, helium–neon laser needs some lag time to luminate after its power is turned on, while a stable luminous flux can be obtained in a short time with the semiconductor laser. This feature could be useful in an emergency laboratory. Other important advantages, especially for smaller laboratories, are its small size and low cost.

Using a low-power semiconductor laser, a clear separation of each subpopulation of white blood cells was obtained. On a scattergram, the gates could be set on clearly distinguishable populations. This paper shows that the precision and linearity of the SF-3000 are good. During storage, the clusters of each subpopulation of white blood cells on the scattergram appeared to enlarge and their density decreased 12 h after blood collection. However, such changes caused only small fluctuations in the differentiation among the clusters, since the values from the stored blood did not significantly differ from those with fresh blood. The changes in the differential counts of the smaller subpopulations may appear large, but the actual differences in the differential counts of such cells were not significant. The effects of the storage temperature were also small. These results were comparable to or even better than, those from other instruments [5, 6]. The white blood cell differential counts from the SF-3000 were compared with the authors' routine method, and the results showed satisfactory correlations.

The SF-3000 was developed for use in smaller laboratories because of its size and the cost. Since it can perform white blood cell differentials, it can save labour and time for smaller clinical laboratories. The analyser has a cap-piercing system for safety. For easy maintenance, control materials have a bar code, a floppy disk can be used to input the values, and control programs have been prepared. Considering these characteristics, the SF-3000 should be useful in those smaller laboratories which do not have haematology experts on the staff, as well as in larger laboratories.

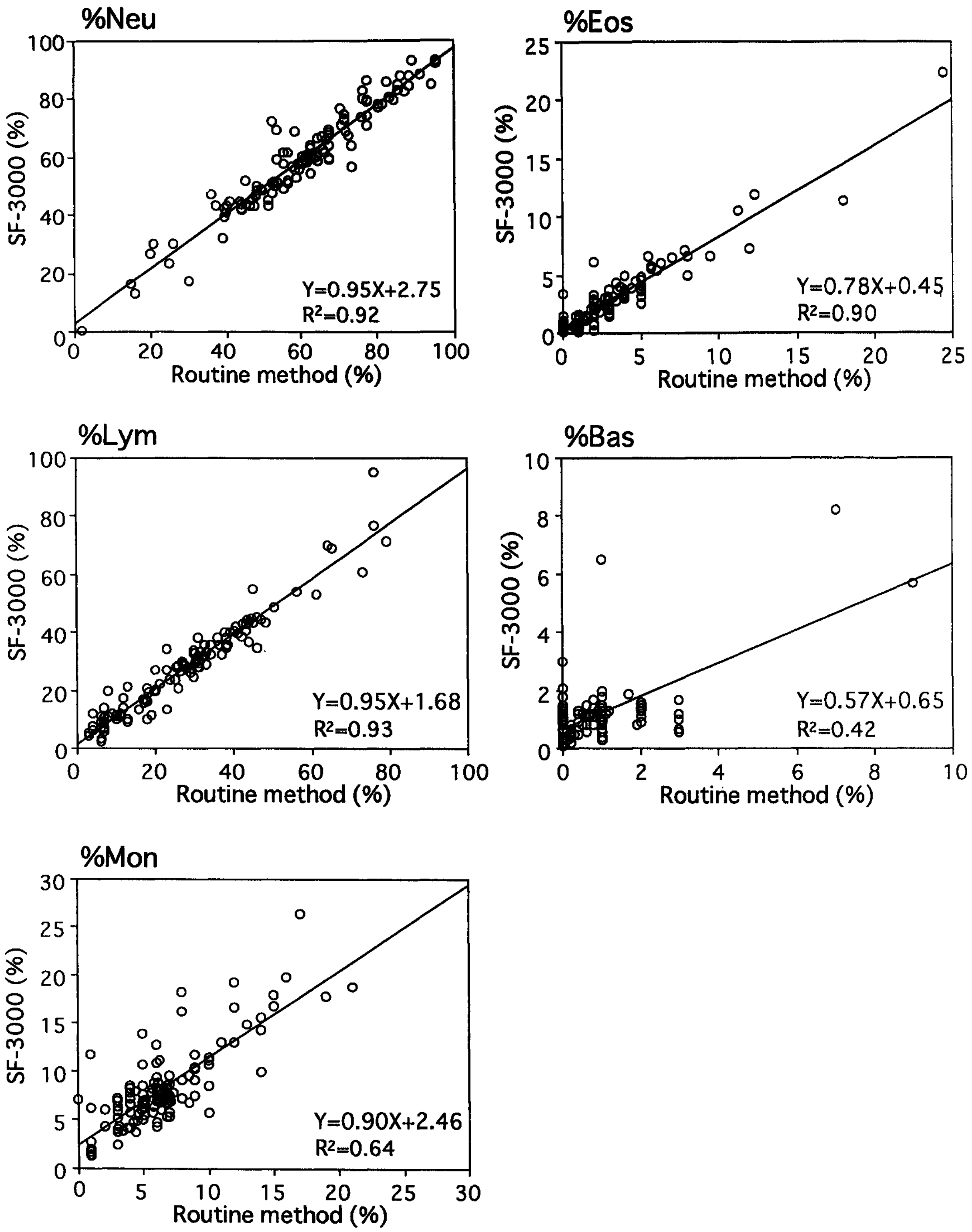

*Figure 6. Correlations between the SF-3000 and the authors' routine method for white blood cell differentials ($N = 118$).*

### References

1. Houwen, B., Chang, L., Chen, W., Clement, L., Ilull, B., Lebeck, L. K. and Mast, B. J., *Sysmex Journal International*, **4** (1994), 5.
2. Karson, A., Maclaren, I., Conn, D. and Wadsworth, L., *American Journal of Clinical Pathology*, **100** (1993), 23.
3. Teratappen, L. W. M. M., deGrooth, B. G., Visscher, K., van Kouterik, F. A. and Greve, J., *Cytometry*, **9** (1988), 39.
4. de Cresce, R., *Laboratory Medicine*, **17** (1986), 17.
5. Sheridan, B. L., Lollo, M., Howe, S. and Bergeron, N., *Clinical Laboratory Haematology*, **16** (1994), 117.
6. Warner, B. A. and Reardon, D. M., *American Journal of Clinical Pathology*, **95** (1991), 20.

